# Estimation of HIV incidence and its trend in three key populations in Iran

**DOI:** 10.1371/journal.pone.0207681

**Published:** 2018-11-29

**Authors:** Hamid Sharifi, Ali Mirzazadeh, Mostafa Shokoohi, Mohammad Karamouzian, Razieh Khajehkazemi, Soodabeh Navadeh, Noushin Fahimfar, Ahmad Danesh, Mehdi Osooli, Willi McFarland, Mohammad Mehdi Gouya, Ali Akbar Haghdoost

**Affiliations:** 1 HIV/STI Surveillance Research Center, and WHO Collaborating Center for HIV Surveillance, Institute for Futures Studies in Health, Kerman University of Medical Sciences, Kerman, Iran; 2 Department of Epidemiology & Biostatistics, University of California San Francisco, San Francisco, CA, United States of America; 3 Institute for Global Health Sciences, University of California, San Francisco, CA, United States of America; 4 Epidemiology and Biostatistics, Schulich School of Medicine & Dentistry, The University of Western Ontario, London, Canada; 5 School of Population and Public Health, Faculty of Medicine, University of British Columbia, Vancouver, BC, Canada; 6 Department of Epidemiology and Biostatistics, School of Public Health, Tehran University of Medical Sciences, Tehran, Iran; 7 Golestan University of Medical Sciences, Department of Health and Social Medicine, Gorgan, Iran; 8 Department of Translational Sciences, Faculty of Medicine, Lund University, Malmö, Sweden; 9 Iran University of Medical Sciences, Tehran, Iran; 10 Modeling in Health Research Center, Institute for Futures Studies in Health, Kerman University of Medical Sciences, Kerman, Iran; University of New South Wales, AUSTRALIA

## Abstract

In Iran, People Who Inject Drugs (PWID), Female Sex Workers (FSW), and prisoners are the main key populations at risk of HIV infection. This study aimed to evaluate the trend of HIV incidence among PWID, FSW and prisoners as an impact measure of HIV harm reduction and prevention efforts in Iran. Data were obtained from the two rounds of national bio-behavioral surveillance surveys among FSW (2010 (n = 872), 2015 (n = 1339)), PWID (2010 (n = 2417), 2014 (n = 2307)), and prisoners (2009 (n = 4536), 2013 (n = 5390)) through facility-based (FSW and PWID surveys) and cluster sampling (prisoner surveys). Time-at-risk was calculated assuming the age at first sex or drug injection as the beginning of the at-risk period and the age at the time of the interview or date when they received a positive HIV test result as the end of this period, adjusted for interval censoring. HIV incidence among PWID in 2014 was 5.39 (95% CI 4.71, 6.16) per 1,000 person-years (PY), significantly lower than in 2009 (17.07, 95% CI 15.34, 19.34). Similarly, HIV incidence was 1.12 (95% CI 0.77, 1.64) per 1,000 PY among FSW in 2015, a significant drop from 2010 (2.38, 95% CI 1.66, 3.40). Also, HIV incidence decreased among prisoners from 1.34 (95% CI: 1.08, 1.67) in 2009 to 0.49 (95% CI: 0.39, 0.61) per 1,000 PY in 2013. Our findings suggest that after an increase in the 2000s, the HIV incidence may have been decreased and stabilized among key populations in Iran.

## Introduction

Iran has a concentrated epidemic, the largest epidemic of HIV in the Middle East [1], with an HIV prevalence of 13.8% (2014) to 15.4% (2010) [2, 3] among people who inject drugs (PWID), 2.1% (2015) to 4.5% (2010) [4, 5] among female sex workers (FSW), and 2.1% (2009) to 1.4% (2013) [6, 7] among prisoners. So far, we have no data on HIV prevalence among other key populations such as men who have sex with men and transgender living in Iran. While most HIV transmissions in Iran (67.2%) have been attributed to injecting drug use, there has been an apparent recent shift to sexual transmission of the virus [8, 9]. An estimated around 75,700 people are living with HIV in Iran, around one-third of them have been diagnosed by 2015[8, 10].

HIV prevention interventions in Iran include the treatment of HIV and diverse sexual and injection harm reduction programs catered towards key populations. HIV testing is available through voluntary testing and counseling centers, drop in centers (DIC), and recently via established but limited number of mobile clinics. Needle/syringe exchanges, opioid substitution therapy, and substance use treatment are provided to PWID. Condom promotion and distribution, sexually transmitted infection (STI) treatment and reproductive health education are available for FSW as well as other key populations [8, 11, 12]. To assess the impact of these prevention interventions among targeted key populations, Iran has conducted several Integrated Bio-Behavioral Surveillance (IBBS) surveys among FSW (2010, 2015), prisoners (2009, and 2013) and PWID (2010, 2014). While these cross-sectional surveys provide estimates of HIV prevalence, the more useful and direct indicator to measure the impact of HIV prevention and treatment programs is HIV incidence.

Measuring the HIV incidence in settings with concentrated epidemic like Iran is challenging as the traditional way of measuring incidence, i.e. prospective cohort studies [13], are expensive, time-consuming, and not nationally representative. While laboratory-based incidence assays [14–16] have improved over the past decade, they require representative samples and complicated algorithm to provide a reliable estimate of the HIV incidence at national levels in settings with concentrated epidemics. Moreover, mathematical modeling methods [17] aimed at calculating the HIV incidence require reliable local input parameters which are often unavailable [18].

In this paper, we used a practical method developed by Osmond *et al*. [19] to estimate the HIV incidence from several national IBBS cross-sectional surveys. We estimated overall and subgroup HIV incidence rate per 1,000 person-years among FSW, PWID, and prisoners using the time since their first sex or injection practice. We also assessed the temporal trends between survey rounds and characterized risk factors associated with higher or lower HIV incidence.

## Methods

We used cross-sectional IBBS surveys data to approximate the incidence of HIV among FSW (2010, 2015), PWID (2010, 2014) and prisoners (2009, 2013) in Iran. In each survey, data were collected on demographics and HIV risk behaviors using face-to-face interview-based questionnaires. To reduce the chance of sensitive data leakage and improve data confidentiality, participants provided verbal consents for the interview and HIV testing. After the interview, if consenting separately, participants were also tested for HIV. In both rounds of FSW and PWID surveys, participants were recruited from facilities (DICs and non-governmental organizations (NGOs) targeting PWID or FSW, counseling centers for vulnerable women, and outreach services targeting FSW or PWID). For the survey among prisoners, we used a multistage cluster random sampling method to recruit eligible participants. All FSW and PWID surveys’ protocols and procedures were reviewed and approved by Kerman University of Medical Sciences IRB Committee before implementation. In both prisoners’ survey, no money or any privileges were given as that may be coercive. In addition to Kerman University of Medical Sciences IRB approval, the Iran Prisons Organization (a prisoner advocacy group) also reviewed and approved the protocols and procedures for the surveys among prisoners. The verbal consent procedure was approved by the ethics committees. To avoid only record linking the subject and the research and keep the study fully anonymous, verbal consent was collected and the research staff signed the consent agreement on behalf of the participants. The research stuff explained to the participants in simple language the aims, risk and benefits of participating in the survey. Upon their verbal agreement, the interview was conducted and blood samples were collected for HIV testing.

### FSW surveys

In the first round of FSW IBBS (2010), we recruited 872 FSW (93.7% consented and provided blood for HIV testing) from 13 cities. Details of survey methods have been presented elsewhere[5]. HIV was tested by ELISA test on two different dried blood samples and those tested positive with both tests were considered as HIV-positive. In the second round (2015), we recruited 1,372 FSW (1339; 97.6% consented and provided blood for HIV testing) from the same 13 cities [4]. In this round, we used two serial rapid tests (Alere and Unigold) to diagnose HIV. We also asked participants about their previous HIV test results and replaced the date of first HIV-positive test with the self-reported date. In FSW survey 2015, the inclusion criteria were a) 18 years or older, b) having penetrative sexual contact (vaginal or anal) for money, drugs, goods or any other favor in last 12 months with more than one man, and c) having Iranian nationality. In FSW survey 2010, we had an additional criterion as d) a history of practicing sex work for at least 6 months.

### PWID surveys

The first round of PWID IBBS recruited 2480 (2418 men and 62 women) participants from 10 cities in 2010, 2,417 (97.5%) of whom consented and provided blood for HIV testing. Details of the study have been published elsewhere[3]. In PWID IBBS 2014, we recruited 2,491 (2,333 men and 58 women) from the same ten cities. Overall, 2,307 (96.1%) agreed and provided blood for HIV testing [2]. In both rounds, HIV was tested by ELISA tests on two different dried blood samples. Both PWID surveys had similar inclusion criteria: a) 18 year or older, b) self-reported drug injection in previous 12 months before the interview, and c) having Iranian nationality.

### Prisoner surveys

The first round of prisoners IBBS recruited 5,530 participants from 27 prisons between May and July 2009, of whom 4,536 (82.0%) consented and provided blood for HIV testing. Details of the study have been published elsewhere[6]. In the second round of the survey (2013), 5,511 prisoners were recruited, 5,390 (97.8%) of whom agreed and provided blood for HIV testing [7]. In both rounds, HIV was tested by ELISA tests on two different dried blood samples. Those tested positive with both tests were considered as HIV-positive. Similar eligibility criteria were used for the two rounds: a) 18 year or older, b) be in prison at least one week before the interview, and c) having Iranian nationality.

### Statistical analysis

We used the age of first sex or drug injection practice (whatever was earlier) as the starting point for the ‘time at risk’ period and the age at the time of interview (or age at first HIV-positive test in FSW IBBS 2015) as the last time point for being at risk. For those who tested HIV positive, we assumed that they have been infected at the mid-point of their time at risk. All participants with HIV-negative result were censored at the time of interview. We calculated the HIV incidence using the method of Osmond et al. [19–21].

$$HIV\;Incidence\;Rate=\frac{Number\;of\;HIV\;infections}{Current\;age‑Age\;at\;first\;sex\;or\;drug\;injection}*1000$$

The point and 95% confidence intervals for the HIV incidence rates were calculated using Kaplan–Meier survival function. We calculated the overall and subgroup HIV incidence rate in all surveys and assessed the differences (temporal trends) in HIV incidence rate between the two rounds using Log-Rank test in Stata v. 14.

We imputed the age at first sex in 2013 prisoner survey as it was not measured directly. We merged the two prisoners’ surveys (2009 and 2013) datasets and ran a multivariate imputation by chained equation (MICE) procedure to impute the age at first sex for all records in 2013. The factors that we used in the imputation model included age at the time of interview, sex, marital status, education, occupation before incarceration, history of drug use, history of drug injection, and age at first drug use.

## Results

### Overall HIV incidence rates

**Fig 1:**
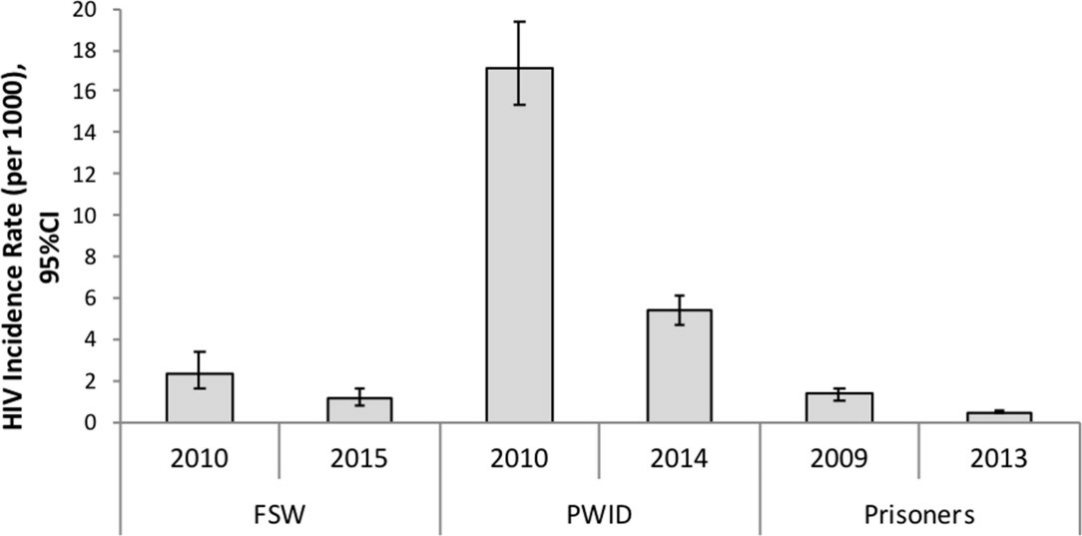
Estimated HIV incidence rate per 1,000 person years among female sex workers (FSW), people who inject drugs (PWID) and prisoners, Iran.

The overall incidence of HIV among FSW decreased from 2.38 (95% CI 1.66, 3.40) per 1,000 person years (PY) from 2010 to 1.12 (95% CI 0.77, 1.64) in 2015 (p-value = 0.003) (Fig 1). HIV incidence also decreased in PWID from 17.07 (95% CI 15.34, 19.34) per 1,000 PY in 2009 to 5.39 (95% CI 4.71, 6.16 in 2014 (p-value<0.001). In contrast, HIV incidence among prisoners also decreased from 1.34 (95% CI 1.08, 1.67) per 1,000 PY in 2009 to 0.49 (95% CI 0.39, 0.61) in 2013 (p-value<0.001) (Fig 1).

### HIV incidence correlates and temporal trends in FSW

Among FSW, HIV incidence measured in the 2010 data was significantly higher for those who reported a history of drug injection (4.72 vs. 1.81 per 1,000 PY, p-value 0.001, Table 1). The same increased HIV incidence was also seen in the 2015 data (4.11 vs. 1.10 per 1,000 PY, p-value 0.001), along with an association with any drug use (1.43 vs. 0.58 per 1,000 PY, p-value 0.04) and also alcohol use (1.59 vs. 0.50 per 1,000 PY, p-value = 0.01). As seen in Table 1, no other significant correlates were significantly associated with elevated HIV incidence among FSW in both FSW surveys. However, HIV incidence decreased in nearly all subgroups of FSW defined by demographic characteristics and risk behaviors.

**Table 1:** Incidence of HIV per 1,000 person-years (PY), female sex workers, Iran, 2010 and 2015.

Variable	2010			2015			p-value for 2010 vs. 2015
	Cases/PY	Rate per 1,000 PY (95% CI)	p-value	Case/PY	Rate per 1,000 PY (95% CI)	p-value	
**Overall**	30/12,606	2.38 (1.66; 3.40)	—	27/24,020	1.12 (0.77; 1.64)	—	0.003
**Age in years**							
<35	17/5,243	3.24 (2.02; 5.22)	0.19	11/7,191	1.53 (0.85; 2.76)	0.83	0.026
≥35	13/7,364	1.77 (1.03; 3.04)		16/16,829	0.95 (0.58; 1.55)		0.05
**Age at first sex, years**							
<15	11/5,337	2.06 (1.14; 3.72)	0.59	10/7,965	1.26 (0.68; 2.33)	0.37	0.13
≥15	19/7,269	2.61 (1.67; 4.10)		17/16,055	1.06 (0.66; 1.70)		0.004
**Current marital status**							
Single	19/7,556	2.51 (1.60; 3.94)	0.58	21/16,143	1.30 (0.85; 2.00)	0.21	0.09
Married	14/4,994	2.20 (1.22; 3.98)		6/7,835	0.77 (0.34; 1.70)		0.005
**Education, years**							
<6	16/7,078	2.26 (1.38; 3.69)	0.95	15/10,866	1.38 (0.83; 2.29)	0.13	0.09
≥6	14/5,528	2.53 (1.50; 4.28)		12/13,295	0.91 (0.52; 1.61)		0.005
**Anal sex history**							
Yes	—	—	—	14/10,724	1.31 (0.77; 2.20)	0.61	—
No	—	—		13/13,295	0.98 (0.57; 1.68)		—
**STI in last year**							
Yes	17/6,170	2.76 (1.71; 4.43)	0.52	20/14,470	1.38 (0.89; 2.14)	0.2	0.02
No	13/6,187	2.10 (1.22; 3.62)		7/9,550	0.73 (0.35; 1.54)		0.01
**Incarceration history**							
Yes	—	—	—	10/6,969	1.43 (0.77; 2.67)	0.42	—
No	—	—		5/2,072	2.41 (1.00; 5.80)		—
**Drug use history**							
Yes	23/9,759	2.36 (1.57; 3.55)	0.88	22/15,395	1.43 (0.94; 2.17)	0.04	0.05
No	6/2,839	2.11 (0.95; 4.71)		5/8,612	0.58 (0.24; 1.39)		0.02
**Drug injection history**							
Yes	10/2,117	4.72 (2.54; 8.87)	0.01	7/1,704	4.11 (1.96; 8.62)	0.001	0.4
No	19/10,480	1.81 (1.16; 2.84)		15/13,661	1.10 (0.66; 1.82)		0.07
**Alcohol use history**							
Yes	14/6,296	2.22 (1.32; 3.75)	0.71	22/13,878	1.59 (1.04; 2.41)	0.01	0.16
No	16/6,299	2.54 (1.56; 4.15)		5/10,098	0.50 (0.21; 1.19)		<0.001

### HIV incidence correlates and temporal trends in PWID

As measured in the 2010 survey wave, HIV incidence was significantly higher among younger PWID (24.08 vs. 12.98 per 1,000 PY, p-value = 0.002) and PWID with a history of incarceration (18.68 vs. 9.15 per 1,000 PY, p-value<0.001) (Table 2). In 2014, HIV incidence was significantly higher among female PWID (16.47 vs. 5.15 per 1,000 PY, p-value<0.001), currently single PWID (6.37 vs. 1.78 per 1,000 PY, p-value<0.001), those with no history of any sex (24.27 vs. 3.56 per 1,000 PY, p-value<0.001), PWID with a history of STI in last year (6.99 vs. 1.08 per 1,000 PY, p-value = 0.001), and those with no history of alcohol use (9.76 vs. 4.12 per 1,000 PY, p-value<0.001). HIV incidence decreased significantly since 2010 in all sub-groups except in females, PWID who started injection at a younger age, and those with no history of sexual contact.

**Table 2:** Incidence of HIV per 1,000 person-years (PY), people who inject drugs, Iran, 2009 and 2014.

	2010			2014			p-value for 2010 vs. 2014
Variable	Cases/PY	Rate per 1,000 PY (95% CI)	p-value	Cases/PY	Rate per 1,000 PY (95% CI)	p-value	
**Overall**	335/19,621	17.07 (15.34; 19.34)	—	213/39,522	5.39 (4.71; 6.16)	—	<0.001
**Age group in years**							
<35	172/7,142	24.08 (20.74; 27.97)	0.002	67/10,799	6.20 (4.88; 7.88)	0.74	<0.001
≥35	162/12,479	12.98 (11.13; 15.14)		146/28,724	5.08 (4.32; 5.98)		<0.001
**Sex**							
Male	329/19,021	17.30 (15.53; 19.27)	0.31	199/38,672	5.15 (4.48; 5.91)	<0.001	<0.001
Female	6/600	10.00 (4.49; 22.26)		14/850	16.47 (9.75; 27.81)		0.16
**Age at first drug use**							
<15	61/3,687	16.55 (12.87; 21.27)	0.93	32/6371.5	5.02 (3.55; 7.10)	0.4	<0.001
≥15	270/15,802	17.09 (15.17; 19.25)		180/32847.5	5.48 (4.79; 6.27)		<0.001
**Age at first injection**							
<15	5/508.5	9.83 (4.09; 23.62)	0.38	3/690	4.35 (1.40; 13.49)	0.62	0.14
≥15	325/19005.24	17.1 (15.34; 19.06)		210/38,326	5.48 (4.79; 6.27)		<0.001
**Current marriage status**							
Single	250/13,739	18.20 (16.08; 20.60)	0.12	198/31,053	6.38 (5.55; 7.33)	<0.001	<0.001
Married	85/5,882	14.45 (11.68; 17.87)		15/8,436	1.78 (1.07; 2.95)		<0.001
**Education, years**							
<6	102/6,673	15.29 (12.59; 18.56)	0.35	74/13,907	5.32 (4.24; 6.68)	0.77	<0.001
≥6	233/12,898	18.06 (15.89; 20.54)		139/25,577	5.43 (4.60; 6.42)		<0.001
**Ever had sex**							
Yes	278/16,969	16.38 (14.57; 18.43)	0.08	128/35,999	3.56 (2.99; 4.23)	<0.001	<0.001
No	57/2,622	21.74 (16.77; 28.19)		85/3,503	24.27 (19.62; 30.2)		0.26
Ever same sex history*							
Yes	38/2,380	15.97 (11.62; 21.94)	0.68	23/5,469	4.21 (2.79; 6.33)	0.44	<0.001
No	233/14,246	16.36 (14.38; 18.60)		99/29,801	3.32 (2.73; 4.05)		<0.001
**STI in last year**							
Yes	37/1,810	20.44 (14.81; 28.22)	0.28	23/3,289	6.99 (4.65; 10.52)	0.001	<0.001
No	285/17,188	16.58 (14.76; 18.62)		3/2,777	1.08 (0.35; 3.35)		<0.001
**Incarceration history**							
Yes	302/16,163	18.68 (16.69; 20.91)	<0.001	179/31,590	5.67 (4.89; 5.56)	0.1	<0.001
No	31/3,386	9.15 (6.44; 13.02)		34/7,894	4.31 (3.08; 6.03)		<0.001
**Alcohol use history**							
Yes	—	—	—	126/30,590	4.12 (3.46; 4.90)	<0.001	—
No	—	—		86/8,809	9.76 (7.90; 12.06)		—

*Only in men

### HIV incidence correlates and temporal trends in prisoners

In 2009, HIV incidence was significantly higher among younger prisoners (2.06 vs. 0.86 per 1,000 PY, p-value = 0.01), male prisoners (1.37 vs. 0.81 per 1,000 PY, p-value = 0.01), prisoners with a previous history of incarceration (1.77 vs. 0.81 per 1,000 PY, p-value<0.003, those reporting not having sex (18.79 vs. 1.20 per 1,000 PY, p-value<0.0001), married prisoners (2.76 vs. 0.64 per 1,000 PY, p-value<0.001), those with a history of drug use (1.63 vs. 0.30 per 1,000 PY, p-value<0.001), those who started drug use at a younger age (3.01 vs. 1.45 per 1,000 PY, p-value = 0.026), prisoners reporting drug injection (4.66 vs. 0.65 per 1,000 PY, p-value<0.001), those who started injection at a younger age (14.62 vs. 4.47 per 1,000 PY, p-value = 0.006), and prisoners with a tattoo (2.26 vs.0.60 per 1,000 PY, p-value<0.0001) (Table 3). In 2013, the HIV incidence was significantly higher among less educated prisoners (0.67 vs 0.49 per 1,000 PY, p-value = 0.001), those with the previous incarceration history (0.65 vs 0.20 per 1,000 PY, p-value<0.001), those with no history of sex (0.97 vs 0.43 per 1,000 PY, p-value = 0.02), married prisoners (0.84 vs 0.22 per 1,000 PY, p-value<0.001), male with a history of same sex behavior (0.95 vs 0.38 per 1,000 PY, p-value = 0.006), those with a history of alcohol drinking (0.58 vs 0.23 per 1,000 PY, p-value = 0.01), those started drug use before 15 (1.03 vs 0.45 per 1,000 PY, p-value = 0.009), those with a history of injection (1.69 vs 0.19 per 1,000 PY, p-value<0.001) and those with a history of tattoo (0.76 vs 0.23 per 1,000 PY, p-value<0.001). HIV incidence decreased significantly from 2009 to 2013 in nearly all demographic and risk subgroups examined, with the exception that no significant change was seen among female prisoners, those started sexual activity before 15, those with a history of STI in last year and those with no history of drug use.

**Table 3:** Incidence of HIV per 1,000 person-years (PY), prisoners, Iran, 2009 and 2013.

	2009			2013			p-value for 2009 vs. 2013
Variable	Case/PY	Rate per 1,000 PY (95%CI)	p-value	Case/PY	Rate per 1,000 PY (95%CI)	p-value	
**Overall**	81/60,447	1.34 (1.08; 1.67)	—	77/157,173	0.49 (0.39; 0.61)	—	<0.001
**Age group in years**							
<35	50/24,324	2.06 (1.56; 2.71)	0.01	33/65,224	0.51 (0.36; 0.71)	0.29	<0.001
≥35	31/36,123	0.86 (0.60; 1.22)		44/91,949	0.48 (0.36; 0.64)		0.01
**Sex**							
Male	78/56,753	1.37 (1.10; 1.72)	0.01	77/154,308	0.50 (0.40; 0.62)	0.24	<0.001
Female	3/3,693	0.81 (0.26; 2.52)		0/2,865	0		0.18
**Education, years**							
<6	42/30,475	1.38 (1.02; 1.86)	0.4	49/73,278	0.67 (0.51; 0.88)	0.001	<0.001
≥6	39/29,871	1.31 (0.95; 1.79)		28/83,698	0.49 (0.39; 0.61)		<0.001
**Previous incarceration**							
Yes	59/33,306	1.77 (1.37; 2.29)	0.003	66/100,878	0.65 (0.51; 0.83)	<0.001	<0.001
No	22/27,141	0.81 (0.53; 1.23)		11/56,241	0.20 (0.11; 0.35)		<0.001
**Ever had sex**							
Yes	72/59,965	1.20 (0.95; 1.51)	<0.001	63/142,516	0.44 (0.35; 0.57)	0.02	<0.001
No	9/479	18.79 (9.78; 36.11)		14/14,458	0.97 (0.57; 1.63)		<0.001
**Age at first sex**							
<15	6/6,567	0.91 (0.41; 2.03)	0.61	7/16,230	0.43 (0.20; 0.90)	0.77	0.19
≥15	63/53,267	1.18 (0.92; 1.51)		56/126,217	0.44 (0.34; 0.58)		<0.001
**Current Marriage Status**							
Single	26/40,408	0.64 (0.44; 0.95)	<0.001	19/88,338	0.22 (0.14, 0.34)	<0.001	<0.001
Married	55/19,943	2.76 (2.12; 3.59)		58/67,755	0.84 (0.65; 1.09)		<0.001
Ever had same sex*							
Yes	—	—	—	15/15,750	0.95 (0.57; 1.58)	0.006	—
No	—	—		47/122,810	0.38 (0.29; 0.51)		—
**STI in last year**							
Yes	5/3,392	1.47 (0.61; 3.54)	0.87	9/10,559	0.85 (0.44; 1.64)	0.09	0.34
No	76/57,016	1.33 (1.06; 1.67)		65/142,148	0.46 (0.36; 0.58)		<0.001
**Alcohol use history**							
Yes	—	—	—	66/113,756	0.58 (0.46; 0.74)	0.01	—
No	—	—		10/43,310	0.23 (0.12; 0.43)		—
**Ever had drug use**							
Yes	77/47,126	1.63 (1.31; 2.04)	<0.001	74/144,341	0.51 (0.41; 0.64)	0.24	<0.001
No	4/13,321	0.30 (0.11; 0.80)		3/12,790	0.23 (0.08; 0.73)		0.77
**Age at first drug use**							
<15	16/5,318	3.01 (1.84; 4.91)	0.026	17/16,445	1.03 (0.64; 1.66)	0.009	0.003
≥15	60/41,286	1.45 (1.13; 1.87)		57/127,896	0.45 (0.34; 0.58)		<0.001
**Ever injected drugs**							
Yes	54/11,591	4.66 (3.57; 6.08)	<0.001	53/31,394	1.69 (1.29; 2.21)	<0.001	<0.001
No	23/35,415	0.65 (0.43; 0.98)		21/112,660	0.19 (0.12; 0.29)		<0.001
**Age at first injection**							
<15	5/342	14.62 (6.09; 35.12)	0.006	1/484	2.07 (0.29; 14.67)	0.94	0.05
≥15	49/10,961	4.47 (3.38; 5.91)		52/30,616	1.70 (1.29; 2.23)		<0.001
**Ever had tattoo**							
Yes	61/26,936	2.26 (1.76; 2.91)	<0.001	59/77,205	0.76 (0.59; 0.99)	<0.001	<0.001
No	20/33,490	0.60 (0.39; 0.93)		18/79,836	0.23 (0.14; 0.36)		<0.003

*Men only

## Discussion

Our findings suggest that HIV incidence has substantially decreased in FSW, PWID and prisoner population in Iran. As a practical approach to approximating incidence rates, our study is the first to assess the magnitude and trends in HIV incidence in three key populations at risk in Iran. The PWID had the highest estimated rate of HIV incidence. We further found that among the FSW and prisoners in our surveys, those with a history of drug injection also had the highest incidence. Identifying injection as the major mode of HIV transmission is also supported by the proportion of national HIV reported cases that could be likely from high-risk injection (67.2% in 2015) [8] and also a recent modeling exercise [8, 22].

Previous studies that estimated HIV incidence from repeated cross-sectional surveys were conducted in African countries with generalized epidemics using household surveys and not particularly assessed HIV incidence among FSW [23, 24]. To the best our knowledge, no study from countries in Middle East and North Africa measured the incidence of HIV among key populations. One study in China, a country with a concentrated epidemic, estimated the HIV incidence as 0.3, 0.6, <0.01 and 0.1 per 1000 persons from 2011 to 2014, respectively [25], slightly lower than our estimates of HIV incidence. In a multisite cohort study of FSW in the Caribbean where HIV prevalence in adults was about 1.0% and 6.1% among adults and FSW, respectively [17] the HIV incidence among FSW was estimated as 0.50% (95% CI 0.06 to 1.81) in the Dominican Republic and 0.39% (95% CI 0.01 to 2.19) in Puerto Rico [13]. As expected, our estimates are in range of previous studies but overall lower than such HIV incidence estimates as in most Caribbean countries where the main modes of transmission is heterosexual and transactional sex. Our estimates are much closer to estimates for FSW in Puerto Rico, where unsafe injection also contributed significantly to the spread of HIV [26].

In PWID, the incidence was lower than the US and higher than Russian PWID. In the US, HIV among PWID in 22 states was estimated as 22.8 per 100,000 person-years [27]. In a cohort study in St Petersburg, Russia, the incidence of infection was estimated as 4.5 per 100 person-years [28]. Moreover, HIV incidence among prisoners in Rhode Island was estimated as 0% [29].

The reduction in the HIV incidence rate among these populations could possibly be explained by expanding the prevention interventions (e.g., increasing number of harm reduction centers for vulnerable women and PWID, scaling up free harm reduction services such as HIV rapid testing services and increasing coverage of Syringe and Needle Program (NSP) among FSW and PWID [9, 18, 30–33]. Since 1999, the number of health centers that provided opioid substitution (OST) therapy has been increased to 700 in 2007 in Iran. Between 2007 and 2009, the number of OST centers were further increased to 1,600 centers with more than 432,000 patients receiving such services. As reported by 2014, there are 3,373 centers offering OST services. Clearly, this highly accessible and coverage of OST nationwide may contribute to the observed reduction of HIV incidence in Iran [34].

In a mathematical modeling study of HIV transmission among PWID in Kermanshah, Iran [12], the incidence rate of HIV was estimated as 1.02% in PWID with sufficient coverage of NSP, and 4.04% in PWID without sufficient coverage of NSP. The reduction in HIV incidence could be due to ongoing nationwide harm reduction program focused on unsafe injection and its associated harms in place since 2002 [35]. Findings from annual prisoners’ sentinel surveillance between 1999 to 2011 [36] indicated a peak between 2001 to 2005 (about 3%) followed by a gradual decrease to 1.28% in 2011. The reduction in HIV was highly correlated with the coverage of methadone maintenance therapy (MMT) and triangular clinics that provided HIV and STI testing and treatment services to prisoners [36]. Interestingly, we found HIV incidence was significantly higher among prisoners and PWID without a history of sex compared to those who reported lifetime sexual contact. Both marriage and having other type of sexual partners require some sort of good health, economic status and accepted social and educational level. Prisoners and PWID who do not have such requirements not only are less likely to have sexual partners but also at higher risk for HIV [37, 38].

The higher incidence of HIV among younger populations could also be explained by a higher frequency of engaging in high-risk sexual acts such as multiple partnerships, unprotected sex, alcohol use, or unsafe drug injections with multiple injecting partners [9, 10, 31]. Although we could fully assess the age effect, it maybe the effect of risk networks and mixtures; people in younger age groups may have contacts with those in older groups who had a higher HIV prevalence; and so, people in younger groups have more risk of acquiring the HIV infection [39–41]. It could also be an artifact as a result of the way we defined the time at risk. Basically, in younger age groups, we shorten the time at risk since first sex or injection and so with the same number of infected cases, the rate would be higher as the denominator will become smaller. This is one of the limitations to the method used [19] which needs to be considered when interpreting the findings.

We acknowledge additional limitations of this study’s method to approximate HIV incidence. We estimated incidence in cross-sectional surveys using the approach outlined by Osmond *et al*. [19] which calculates person-time at risk since first sex or injection by recall. First, participants may not recall or report the accurate data of first sex or injection. Second, we assumed people remained at risk for HIV after their first sex or injection and did not exclude periods of stopping sexual activity or injection or practicing safe sex or safe injection. Third, the exact date of seroconversion is censored at some time between the true (but unknown) date of sex or injection initiation and the first time they tested for HIV. For our study populations, testing rates are low [9, 31, 42], and the survey itself may be the first test. To partly reduce the effect of such bias, we used the midpoint of time at risk as the time of seroconversion in our analysis. Moreover, the approximated exposure period cannot establish cause before effect, which is the major strength of longitudinally measured HIV incidence.

## Conclusions

Our findings suggest that after an increase in the 2000s, HIV transmission may have decreased and stabilized among PWID, FSW and prisoner populations in Iran. This reduction can be attributed to the nationwide expansion of OST and harm reduction services. However, still injection continues to be the main driver of HIV transmission in Iran. Adding other prevention strategies like pre-exposure prophylaxis (PrEP), strategies to improve early HIV diagnosis, timely identification of HIV transmission clusters by using recency HIV testing options, and using the test and treat strategy to reduce the individual- and population-level viral load may further contribute to the epidemic control and reaching the 90-90-90 targets by 2020.
